# Development of Fish Oil-Loaded Microcapsules Containing Whey Protein Hydrolysate as Film-Forming Material for Fortification of Low-Fat Mayonnaise

**DOI:** 10.3390/foods9050545

**Published:** 2020-04-30

**Authors:** Nor E. Rahmani-Manglano, Irene González-Sánchez, Pedro J. García-Moreno, F. Javier Espejo-Carpio, Charlotte Jacobsen, Emilia M. Guadix

**Affiliations:** 1 Department of Chemical Engineering, University of Granada, 18071 Granada, Spain; norelenarm@ugr.es (N.E.R.-M.); irenegs@correo.ugr.es (I.G.-S.); fjespejo@ugr.es (F.J.E.-C.); eguadix@ugr.es (E.M.G.); 2 Division of Food Technology, National Food Institute, Technical University of Denmark, 2800 Lyngby, Denmark; chja@food.dtu.dk

**Keywords:** omega-3, microencapsulation, spray-drying, whey protein, lipid oxidation, food fortification

## Abstract

The influence of the carbohydrate-based wall matrix (glucose syrup, GS, and maltodextrin, MD21) and the storage temperature (4 °C or 25 °C) on the oxidative stability of microencapsulated fish oil was studied. The microcapsules (ca. 13 wt% oil load) were produced by spray-drying emulsions stabilized with whey protein hydrolysate (WPH), achieving high encapsulation efficiencies (>97%). Both encapsulating materials showed an increase in the oxidation rate with the storage temperature. The GS-based microcapsules presented the highest oxidative stability regardless of the storage temperature with a peroxide value (PV) of 3.49 ± 0.25 meq O_2_/kg oil and a content of 1-penten-3-ol of 48.06 ± 9.57 ng/g oil after six weeks of storage at 4 °C. Moreover, low-fat mayonnaise enriched with GS-based microcapsules loaded with fish oil and containing WPH as a film-forming material (M-GS) presented higher oxidative stability after one month of storage when compared to low-fat mayonnaise enriched with either a 5 wt% fish oil-in-water emulsion stabilized with WPH or neat fish oil. This was attributed to a higher protective effect of the carbohydrate wall once the microcapsules were incorporated into the mayonnaise matrix.

## 1. Introduction

Food fortification with omega-3 polyunsaturated fatty acids (ω-3 PUFAs) has gained increased scientific and industrial interest in the last decades [1]. This is mainly due to the health benefits attributed especially to eicosapentaenoic (EPA; C20:5 n-3) and docosahexaenoic (DHA; C22:6 n-3) fatty acids [2,3]. However, the high number of bis-allylic hydrogens present in these ω-3 PUFAs make them highly prone to oxidation, leading to: (i) the loss of their nutritional properties and (ii) the appearance of odor/flavor-active and/or other potentially toxic compounds [4]. Hence, the development of efficient delivery systems which prevent ω-3 PUFAs oxidation prior (e.g., delivery system production); during (e.g., food processing) and after its incorporation into complex food matrices (e.g., food storage) is of great importance. In this regard, encapsulation technologies are of special interest for the food industry, since they allow the design of functional food systems overcoming the inherent drawbacks of ω-3 PUFAs rich oils (e.g., fish oil), such as low oxidative stability, low solubility and oily texture. Encapsulation consists of entrapping the core material (e.g., fish oil) within a homogeneous/heterogeneous matrix (e.g., encapsulating agent/s) to develop a physical barrier between the bioactive compound and the environment, thus preventing its degradation, easing its handling and/or controlling the bioactive release [5]. Among all the available encapsulation techniques (e.g., freeze-drying or coacervation), spray-dying is the most commonly used by the food industry to encapsulate bioactive ingredients [6]. Fish oil microencapsulation by spray-drying has been widely studied over the last decade, being the most commonly used encapsulating agents of carbohydrates, proteins and their combinations [7].

Nonetheless, additional stabilization techniques are required (e.g., addition of antioxidants to the formulation), since it has been demonstrated that the emulsification process and subsequent drying using air at high temperatures (170−200 °C) result in the initial oxidation of the oil [8,9]. Bioactive compounds such as protein hydrolysates or peptides, which exhibit both emulsifying and antioxidants properties, are a promising alternative to conventional antioxidants (e.g., tocopherols). In heterogeneous systems such as fish oil-in-water emulsions (which is the feed to the spray-drier), the location of the antioxidants determines their antioxidant activity [10,11]. The same is also the case for the resulting microcapsule. Antioxidants located at the oil/water interface (in emulsions) or at the oil/encapsulating agent interface (in spray-dried microcapsules) will be preferred to inhibit lipid oxidation. This is because these interfaces are the place where oxidation is started by the contact of prooxidants (e.g., trace of metals and oxygen) and the oil.

Whey protein hydrolysates (WPH) have been reported to exhibit antioxidant activity (e.g., radical scavenging activity, metal chelating and reducing power) [12,13]. Moreover, due their high emulsifying activity, WPH have also been used to produce fish oil microcapsules in combination with maltodextrin (DE 16.5−19.5) [14,15]. Nevertheless, the protective effect of WPH on the reduction of lipid oxidation in the microcapsules was not investigated in any of the latter studies. More recently, Tamm et al. [16] produced fish oil-loaded microencapsulates using glucose syrup as the wall material in the presence of unmodified or hydrolyzed β-lactoglobulin (β-LG). The unmodified and hydrolyzed proteins were used as a film-forming material around the oil droplets. The authors found that β-LG hydrolysates enhanced the oxidative stability of microencapsulated fish oil when compared to the unmodified protein. This fact was attributed to an improved accessibility of the resulting amino acid residues with antioxidant properties after enzymatic hydrolysis. Thus, the results reported by Tamm et al. [17] showed a promising potential for the use of spray-dried microcapsules loaded with fish oil and obtained with WPH as a delivery system of ω-3 PUFAs. However, the enrichment of food products with ω-3 microencapsulates having WPH as a film-forming material, and its effect on the physicochemical properties of the fortified product remains to be evaluated.

In this regard, the aims of the present study were to investigate: (i) the effect of the carbohydrate-based wall material (glucose syrup or maltodextrin) and (ii) the storage temperature (4 °C or 25 °C) on the oxidative stability of spray-dried microcapsules loaded with fish oil and produced using WPH as a film-forming material. In addition, the feasibility of using spray-dried microcapsules containing WPH for fortifying a low-fat mayonnaise (40 wt% of total oil) with ω-3 PUFAs was assayed. For that purpose, the physical and oxidative stabilities of low-fat mayonnaise enriched either with microcapsules loaded with fish oil, a fish oil-in-water emulsion stabilized with WPH or neat fish oil were investigated during one month of storage.

## 2. Materials and Methods 

### 2.1. Materials

Whey protein (34.6 wt% protein content) and maltodextrin (DE 21) were kindly provided by Abbott (Granada, Spain), while the glucose syrup (DE38, C*Dry 1934) was kindly donated by Cargill Germany GmbH (Krefeld, Germany). Alcalase 2.4 L was purchased from Novozymes (Bagsvaerd, Denmark). Refined fish oil (Omega Oil 1812 TG Gold) was acquired from BASF Personal Care and Nutrition GmbH (Illertissen, Germany) and stored at −80 °C until use. The fatty acid composition of the fish oil was determined by gas chromatography (GC), as described in [17], and it was as follows (major fatty acids in %, *w*/*w*): 7.0% myristic acid (C14:0), 16.7% palmitic acid (C16:0), 8.8% palmitoleic acid (C16:1n-7), 4.1% stearic acid (C18:0), 8.2% oleic acid (C18:1n-9), 19.3% eicosapentaenoic acid (C20:5n-3) and 16.1% docosahexaenoic acid (C22:6n-3). Peroxide value (PV) of the fish oil was measured as described in Section 2.5.4 and was 0.36 ± 0.03 meq O_2_/kg oil. Tocopherol content (TC) of the fresh oil was measured as described in Section 2.5.4 and was: 427.0 ± 0.0 µg/g oil, 48.0 ± 1.4 µg/g oil, 1891.0 ± 12.7 µg/g oil and 644.3 ± 4.1 µg/g oil for alpha-, beta-, gamma- and delta-tocopherol, respectively. Refined sunflower oil (SFO), for the production of mayonnaise, was purchased from the local market. The specified fats compositions as given by the supplier were: 10.77 wt% saturated fats, 30.47 wt% monounsaturated fats and 58.76 wt% polyunsaturated fats. Peroxide value (PV) of the sunflower oil was measured as described in Section 2.7.2 and was of 3.5 ± 0.2 meq O_2_/kg oil. Stabilizer Grinsted FF 1149 was kindly donated by DuPont (DuPont Nutrition Biosciences Aps, Haderslev, Denmark). The rest of the ingredients used in the production of the mayonnaises were purchased in the local market. The rest of reagents used for analysis were of analytical grade.

### 2.2. Enzymatic Hydrolysis of Whey Protein 

Enzymatic hydrolysis of whey protein (WP) was carried out in an automatic titrator 718 Stat Titrino (Metrohm AG, Herisau, Switzerland) to a degree of hydrolysis 10% (DH 10) with alcalase. For this purpose, a solution containing 36 g of protein was prepared with distilled water to a final volume of 0.9 L. The process conditions were set to 50 °C and the pH to 8, and the enzyme-substrate ratio was fixed to 0.55 (*w*/*w*). The degree of hydrolysis was estimated with the pH-stat-method, as described by Camacho et al. [18]. The hydrolysis reaction took ca. 1.5 h, and the enzyme was deactivated at 100 °C for 5 min. The whey protein hydrolysate (WPH) solution was stored at −20 °C until further use.

### 2.3. Microencapsulation of Fish Oil by Spray-Drying

Previous to spray-drying, fish oil-in-water emulsions stabilized with WPH and containing one of the encapsulating agents were produced. The aqueous phase of the emulsions was prepared by dissolving the glucose syrup (GS) or maltodextrin (MD21) (28%, *w*/*w*) in the WPH solution and adding distilled water in order to have a protein content of 2 wt%. Then, the pH was adjusted to pH 8. A prehomogenization process was carried out for 2 min at 15,000 rpm using an Ultraturrax T-25 homogenizer (IKA, Staufen, Germany), while the oil (5%, *w*/*w*) was added during the first minute. The coarse emulsions were then homogenized in a high-pressure homogenizer (PandaPLUS 2000; GEA Niro Soavi, Lübeck, Germany) at a pressure range of 450/75 bar, applying 3 passes. The temperature during the emulsification process was kept under 32 °C. Subsequently, the emulsions were dried in a pilot plant scale spray-drier (Mobile Minor; Niro A/S, Copenhagen, Denmark) at 190/80 °C inlet/outlet temperature, respectively. The pressure of the pneumatic air activating the rotary atomizer was set to 4 bar, which implies a rotational speed of the atomizer of 22,000 rpm. 

### 2.4. Oil Droplet Size Distribution (ODSD)

The oil droplet size distribution (ODSD) of both the parent emulsions (emulsions fed to the spray-drier) at day 0 and the reconstituted emulsions (emulsions resulting of redispersing the microcapsules in water) at days 0 (week 0), 14 (week 2), 28 (week 4) and 42 (week 6) were measured by laser diffraction in a Mastersizer 2000 (Malvern Instruments, Ltd., Worcestershire, UK), as described by García-Moreno et al. [19]. The emulsions were reconstituted by dissolving the powder in distilled water in order to achieve the same solids content as the original emulsion. Measurements were made in duplicate, and the results are given in 90th percentile.

### 2.5. Physicochemical Characterization of Microencapsulates 

#### 2.5.1. Moisture Content (MC) and Water Activity (a_w_) 

The moisture content of the microcapsules was determined using an infrared balance (AD-471A, Tokyo, Japan) where ca. 1 g of powder was heated at 105 °C for 90 min until a constant weight. The water activity was measured using a LabMASTER-aw (Novasina AG, Lachen, Switzerland) at 20 °C. The measurements were carried out in duplicates.

#### 2.5.2. Encapsulation Efficiency (EE) 

The encapsulation efficiency (EE) was determined by extracting the surface oil, as described by Danviriyakul et al. [20], with some modifications. In brief, 2.5 g of powder was weighed and mixed with 15 mL of hexane in a vortex mixer for 2 min and then centrifuged at 2720× *g* for 20 min. Five mL of supernatant were collected on a Pyrex tube, previously weighed, and the solvent was evaporated under a constant flow of nitrogen. After total solvent evaporation, in order to calculate the surface oil, the Pyrex was weighed again, and the oil concentration was adjusted to the initial volume of hexane added. The *EE* was calculated as follows:

(1) $$EE,\;\%=\frac{A-B}{A}\times 100$$

where *A* refers to the total theoretical amount of oil (g) and *B* to the non-encapsulated oil (g). Measurements were carried out in triplicate.

#### 2.5.3. Morphology and Size

The morphology and size of the microcapsules were studied by means of scanning electron microscopy (SEM) using a FEI microscope (FEI Inspect, Hillsboro, OR, USA). For this purpose, a thin layer of microcapsules was placed on a carbon tape and sputter-coated with gold, 8 s, 40 mA using a Cressington 208HR Sputter-Coater (Cressington Scientific Instruments, Watford, England). The SEM images were taken in the range 830×–870× magnification with a 10-kV accelerating voltage. Then, they were analyzed using the ImageJ software (National Institute of Health). To determine the mean diameters of the microcapsules, more than 150 randomly selected microcapsules were measured.

#### 2.5.4. Oxidative Stability 

Spray-dried microcapsules loaded with fish oil were stored at 4 °C and 25 °C during 6 weeks in brown bottles (30 mL and 26-mm inner diameter). Each bottle contained 10 g of microcapsules. Samples were taken at days 0 (week 0), 7 (week 1), 14 (week 2), 21 (week 3), 28 (week 4), 35 (week 5) and 42 (week 6) and placed at −80 °C under a nitrogen atmosphere until the determination of the peroxide value (PV), tocopherol content (TC) and content of secondary volatile oxidation products (SVOP) was carried out. 

##### Peroxide Value (PV)

Fish oil was extracted from the microcapsules, as described by Bligh and Dyer [21] using a reduced amount of chloroform/methanol (1:1, *w*/*w*) solvent. For the extraction, ca. 2 g of powder were weighed and then dissolved by adding 10 mL of distilled water. Peroxide value (PV) was then quantified on the lipid extracts using the colorimetric ferric-thiocyanate method at 500 nm, according to Shantha and Decker [22]. In brief, the extracted oil was diluted in chloroform/methanol (7:3, *v*/*v*) prior to the addition of iron-II-chloride and ammonium thiocyanate solutions. Then, the mixture was incubated for 5 min at room temperature. Measurements were carried out in duplicates. Results were expressed in meq O_2_ per kg of oil.

##### Tocopherol Content (TC)

Tocopherol content of the microencapsulated oil was determined by HPLC (Agilent 1100 Series) according to the American Oil Chemists’ Society (AOCS ) official method [23]. In brief, about 2 g of the chloroform extract was evaporated under nitrogen and dissolved in 1-mL n-heptane, and from this, 0.8 mL were taken into separate vials before injection of an aliquot (40 µL) on a Spherisorb S5W column (250 × 4.6 mm) (Phase Separation Ltd., Deeside, UK). Elution was performed with an isocratic mixture of n-heptane/2-propanol (100:0.4, *v*/*v*) at a flow of 1 mL/min. Detection was done using a fluorescence detector with excitation at 290 nm and emission at 330 nm, according to the AOCS [23]. Measurements were performed in duplicate and quantified by authentic standards. Results were expressed in µg tocopherol per g of oil.

##### Secondary Volatiles Oxidation Products (SVOP)—Dynamic Headspace GC-MS

Approximately, 1 g of powder and 30 mg of internal standard (4-methyl-1-pentanol and 30 µg/g water) were weighed in a purge bottle and mixed with 10 mL of distilled water. Then, the bottle content was heated for 30 min in a water bath at 45 °C while purging with nitrogen (flow rate 150 mL/min). The volatile compounds released were retained in Tenax GR tubes (Perkin Elmer 1/4″ stainless steel tubes packed with 225 ± 3 mg Tenax GR 60–80 mesh). Then, these compounds were desorbed again by means of helium and heat (200 °C) in an Automatic Thermal Desorber (ATD-400; Perkin Elmer, Norwalk, CN), cryofocused on a cold trap (−30 °C), released again (220 °C) and led to a gas chromatograph (HP 5890IIA; Hewlett Packard, Palo Alto, CA, USA and Column: DB-1701, 30 m × 0.25 mm × 1.0 µm; J&W Scientific, Folsom, CA, USA). The individual compounds were analyzed by mass-spectrometry (HP 5972 mass-selective detector; Agilent Technologies, Santa Clara, California, USA; electron ionization mode: 70 eV and mass to charge ratio scan between 30 and 250). The released volatile compounds were then identified by MS-library searches (Wiley 138K, John Wiley and Sons, Hoboken, New Jersey, USA and Hewlett-Packard, San Jose, California, USA) and quantified through calibration curves using external standards (butanal, pentanal, 1-penten-3-ol, hexanal, (*E*)-2-hexenal, heptanal, octanal, (*E,E*)-2,4-heptadienal, (*Z*)-4-heptenal and nonanal) dissolved in 96% ethanol. The standard solutions were diluted to concentrations of approximately 2.5, 5, 10, 50, 100 and 500 µg/mL, and 1 µL of each was directly injected on the Tenax tubes. Measurements were made in triplicates for each sample. Results were expressed as ng/g oil. 

### 2.6. Production of Fortified Mayonnaise

Light mayonnaise (40 wt% of total oil) fortified with ω-3 PUFAs (2.5 wt% fish oil) was produced following three different approaches: (i) incorporating neat fish oil (M-NFO), (ii) incorporating a fish oil-in-water emulsion stabilized with WPH (M-EM) and (iii) incorporating spray-dried microcapsules loaded with fish oil and obtained with WPH as a film-forming material and glucose syrup as the encapsulating agent (M-GS). In all cases, 300 g of mayonnaise containing: 2.5 wt% of fish oil, 37.5 wt% of SFO, 4 wt% of egg yolk, 1 wt% of vinegar, 0.4 wt% of lemon juice and 0.3 wt% of salt were prepared as described by Miguel et al. [24], with some modifications. In the case of M-NFO and M-EM samples, 1 wt% of sugar was also added. For the mayonnaise preparation, first, distilled water, salt, sugar and 6 mL of sodium azide solution (0.0125 g/mL) were mixed in a blender (Taurus robot, 300 inox.) for 15 s. For the M-EM sample, also 150 g of emulsified fish oil were added in the first step. Then, the egg yolk was incorporated and mixed for 15 s. Stabilizer Grinsted FF was manually dissolved in 10 g of sunflower oil (and fish oil in the case of the M-NFO sample) and added to the blender (15 s mixing). Then, the rest of the oil was added in three steps (except ca. 10 wt%) and mixed for 30 s each. Vinegar and lemon juice were dispersed manually to the remaining oil (ca. 10 wt%) and added as the last step. Mixing in this case lasted 30 s. In the case of the M-GS sample, 58.5 g of microcapsules were added to the blender at this last step and mixed for 45 s to complete the dispersion. 

### 2.7. Characterization of Fortified Mayonnaise 

#### 2.7.1. Physical Stability: Droplet Size Distribution and Viscosity

The droplet size distribution and viscosity of the fortified mayonnaises were evaluated after production and during 28 days of storage at 25 °C. 

The droplet size distribution was measured as described in Section 2.4. For this purpose, 1 g of mayonnaise was dissolved in sodium dodecyl sulfate (SDS) buffer (10-mM NaH_2_PO_4_, pH 7) to a ratio of 1:5 (*w*/*w*). Then, the solution was sonicated for 15 min to avoid droplets agglomeration. Measurements were made in triplicate, and the results are given in surface area mean diameter (D[3,2]) and volume weighted mean diameter (D[4,3]). 

The viscosity of the fortified mayonnaise samples was measured using a rotatory Kinexus Malvern rheometer (Malvern Panalytical Ltd., Worcestershire, UK) equipped with a plate-plate geometry. An increasing gradient of stress was applied from 0.1–200 Pa at 25 °C. Measurements were made in triplicate. 

#### 2.7.2. Oxidative Stability

To monitor the oxidative stability of low-fat fortified mayonnaise, 40 g of each sample were stored in polyethylene containers (60 mL) at 25 °C during 28 days. Samples were taken at days 0 (week 0), 7 (week 1), 14 (week 2), 21 (week 3) and 28 (week 4) and kept under an inert atmosphere at −80 °C until analysis.

##### Peroxide Value (PV)

Oil extraction from the low-fat mayonnaise for the PV determination was made as follows: ca. 0.5 g of mayonnaise were weighed and mixed with 5 mL of distilled water. Then, 20 mL of hexane/2-propanol (1:1, *v*/*v*) solvent was added and mixed for 5 min. The resulting mixture was centrifuged at 670× *g* for 2 min. PV was measured using the thiocyanate assay, as described in Drusch et al. [25], with some modifications. Extracted oil was diluted with 2-propanol prior to the addition of iron-II-chloride and ammonium thiocyanate solutions and then was incubated for 5 min at 25 °C. Oil extraction was made in duplicate for each sample. The PV measurements were made in triplicate for each oil extract. Results were expressed in meq O_2_ per kg of oil.

##### P-Anisidine Value (AV)

For the p-anisidine value determination, 2.5 g of mayonnaise were weighed to carry out the oil extraction, as described in Section 2.7.2. Oil extraction was made in duplicate for each sample. The AV measurements were made in triplicate according to the ISO 6885:2006 method [26] for each oil extract.

### 2.8. Statistical Analysis

For data analysis, the software Statgraphics Centurion XV (Statistical Graphics Corp., Rockville, MD, USA) was used. First, one-way ANOVA was performed to identify if there were differences between the samples. Then, a multiple sample comparison using Tukey’s test allowed to identify means which were significantly different from each other. Differences between means were considered significant at *p* ≤ 0.05.

## 3. Results

### 3.1. Oil Droplet Size Distribution (ODSD) of Emulsions

The parent emulsions and reconstituted emulsions presented values of the 90th percentile below 2 µm (Table 1), indicating that the WPH efficiently stabilized the oil droplets by maintaining the structural integrity of the oil/water interface prior, during and after the microencapsulation process [16]. Despite the lack of surface-active properties of the encapsulating agents used, the results show significant differences (*p* < 0.05) for the ODSD of the parent emulsions prior to drying, which are mainly attributed to minor differences in pressure adjustments in the homogenizer. In this line, Hogan et al. [27] found that the volume average diameter (D[4,3]) of soy oil emulsions stabilized with sodium caseinate did not differ irrespective of the dextrose equivalence (DE) of the non-surface-active carbohydrate used as a wall constituent.

After drying, a small but significant (*p* < 0.05) increase in the ODSD occurred in both samples at week 0, suggesting coalescence of the non-encapsulated oil after reconstitution [28]. In the case of GS-based reconstituted emulsions, the ODSD during six weeks of storage was in the range 0.61–0.66 µm for both storage temperatures. On the other hand, for MD21-based emulsions, the ODSD increased progressively during the storage time from 0.66 to 0.82 µm at 4 °C and 0.66 to 0.88 µm at 25 °C. These results suggest oil leakage in the MD21 microcapsules, which increased the surface fat content and led to more oil available to coalesce after reconstitution. This finding may be related to the poorer retention properties of MD21 as a wall material when compared to GS, attributed to the lower molecular weight of the oligosaccharides present in the glucose syrup. Moreover, the results show that the storage temperature had little effect on the retention properties of the encapsulating agents used, despite the statistical analysis results showing significant differences for some sampling points for both storage temperatures assayed. 

### 3.2. Physicochemical Characterization of Microencapsulates

#### 3.2.1. Moisture Content, Water Activity and Encapsulation Efficiency (EE)

The moisture content (MC) was of 4.7% ± 0.4% and 4.2% ± 0.1% for the GS and MD21-based microencapsulates, respectively. These low MC values, together with the low water activity (a_w_) of the microencapsulates (GS, 0.184 ± 0.004 and MD21, 0.187 ± 0.001), are desired in order to confer long-term microbiological stability for the microcapsules. Moreover, as no significant differences could be observed between the samples (*p* > 0.05), it can be assumed that the MC and a_w_ are independent of the encapsulating agent used (while maintaining constant the spray-drying processing variables).

High EE values were obtained for both types of microcapsules, although a significantly higher EE value was found for the GS-based microencapsulates when compared to the MD21-based microcapsules (98.07 ± 0.04% vs. 97.66 ± 0.06%). Interestingly, the slightly higher EE value of the GS-based microencapsulates cannot be attributed to a smaller droplet size of the parent emulsion (Table 1). Thus, differences in the EE may be consequence of a positive correlation between the EE of encapsulates and DE of the carbohydrates used as wall materials [27,29,30]. This fact is explained on the basis that increasing the DE of the carbohydrate leads to smaller oligosaccharides, which are thought to form a more uniform and denser packaging of the core material, which favors oil encapsulation.

#### 3.2.2. Morphology and Size

**Figure 1:**
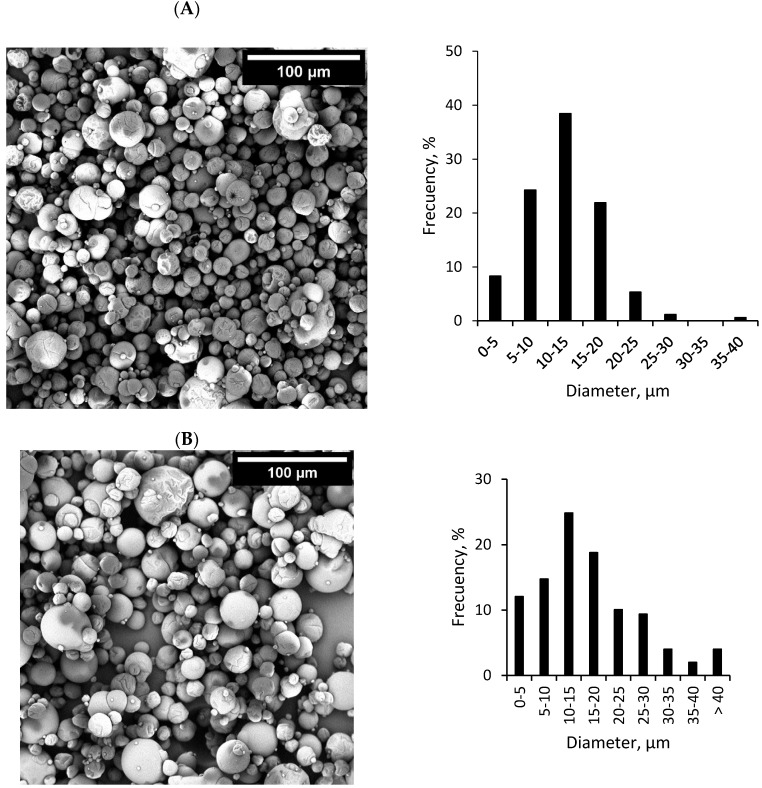
SEM micrographs and diameter distribution of fish oil-loaded microcapsules containing whey protein hydrolysate (WPH) as a film-forming material and glucose syrup (**A**) or maltodextrin DE21 (**B**) as the encapsulating agents.

Figure 1 shows the morphology and particle size distribution of the microcapsules. Both types of microcapsules showed a spherical shape with smooth surfaces, even though a few microcapsules with wrinkled surfaces could also be observed. Furthermore, no particle agglomerations were detected, which can be attributed to the low surface fat content of the microencapsulates produced [27]. Microcapsules prepared with MD21 presented a wider particle size distribution when compared to the microcapsules produced with GS. The percentage of microcapsules with diameters below 20 µm was 92.9% and 70.5% for the GS and MD21-based microencapsulates, respectively (Figure 1), while the particle mean diameters were 12.5 ± 5.5 µm and 16.8 ± 10.2 µm, respectively. Since the rotational speed of the atomizer was kept constant, the larger particle size of the MD21-based microencapsulates was most likely due to the higher viscosity of the MD21-feed emulsion, which led to larger emulsion droplets after atomization and, hence, larger dried microcapsules when compared to the GS-feed emulsion [27,31]. 

#### 3.2.3. Oxidative Stability of the Microencapsulates

##### Peroxide Value (PV) and Tocopherol Content (TC)

**Figure 2:**
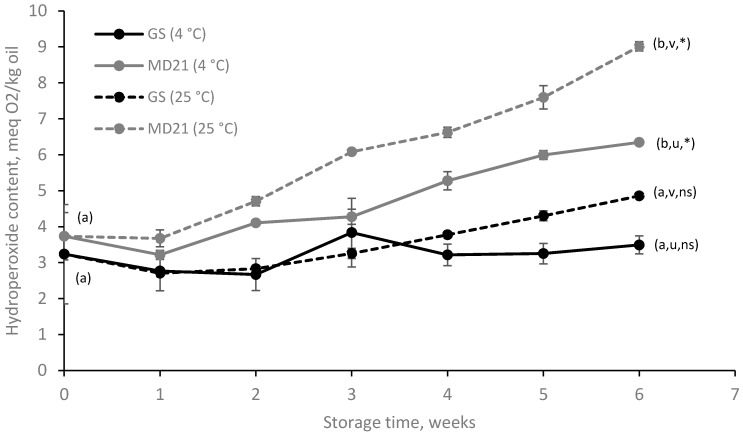
Peroxide value (PV) of spray-dried microcapsules loaded with fish oil during storage at: 4 °C (solid line, —•—) and 25 °C (broken line, - -•- -) encapsulated with glucose syrup (black) or maltodextrin (grey). Means within the same sampling point followed by a letter, a-b, indicates statistical differences (*p* ≤ 0.05) between encapsulating agents for the same storage temperature. Means within the same sampling point followed by a letter, u-v, indicates statistical differences (*p* ≤ 0.05) between storage temperatures for the same encapsulating agent. Means within the same sample followed by an asterisk (*) indicates statistical differences (*p* ≤ 0.05) between week 0 and week 6. Means within the same sample followed by “ns” indicates no statistical differences (*p* > 0.05) between week 0 and week 6.

Oxidative stability of the microencapsulates was first evaluated by determining the formation of the primary oxidation products during storage (Figure 2). Right after production, PV of 3.24 ± 1.38 and 3.73 ± 0.66 meq O_2_/kg oil were obtained for the GS and MD21-based microcapsules, respectively. No significant differences (*p* > 0.05) in the PV were observed between the two types of microcapsules at day 0, although a significant increase on the hydroperoxide content compared to the fresh oil (PV = 0.36 ± 0.03 meq O_2_/kg oil) could be noted. This is attributed to the encapsulation process itself, which involves mechanical stress, shear forces and heat, leading to the initial oil oxidation [8,9]. As expected, lipid oxidation was accelerated for the two types of microcapsules when increasing the storage temperature from 4 to 25 °C. For lipid autoxidation, an increase of 10 °C temperature results in a two-fold increase in the reaction rate [32]. However, the effect was more pronounced in the MD21-based microcapsules (PV = 9.01 ± 0.13 meq O_2_/kg oil after six weeks storage at 25 °C). This positive correlation between the lipid oxidation rate and storage temperature on microencapsulated fish oil has also been reported by other authors [33,34] and could be attributed to the loss of natural antioxidants, as well as an increase in diffusivity of the prooxidant agents (e.g., oxygen, radicals and trace of metals) at higher temperatures. 

Nonetheless, although the temperature influenced lipid oxidation, higher PV values were found in MD21-based microcapsules in all cases. These results indicate that the influence of the wall material (e.g., which determines the permeability to prooxidant species and retention properties) predominated over the storage temperature on microencapsulated fish oil oxidative stability. The lower PV of the GS-based microencapsulates has been attributed to the DE differences between carbohydrates and, thus, the molecular weight. Such differences affect the matrix free volume elements and the oxygen diffusivity through the wall. The latter increases with the increasing molecular weight of the carbohydrate [35,36]. Moreover, the ODSD results showed oil leakage during the storage time for MD21-based microencapsulates (Section 3.1), which resulted in lower EE for MD21-based microcapsules and increased the content of easily oxidizable oil on the surface of these microcapsules. 

Moreover, it is noteworthy that the PV results of this study were lower than others reported in literature that also produced fish oil microencapsulates within carbohydrate matrices. For instance, Drusch and Berg [37] produced fish oil-loaded microcapsules (30 wt% or 50 wt%) by spray-drying into a matrix of nOSA-starch and GS at two drying temperature settings (160/60 °C or 210/90 °C) with PV of ca. 160–340 meq O_2_/kg oil after six weeks of storage at 20 °C. Likewise, Morales-Medina et al. [9] microencapsulated fish oil using fish protein hydrolysates as emulsifiers and GS as the encapsulating material. The PV of the microencapsulates reported by the authors after six weeks of storage at 20 °C were also higher than those values obtained in the current study (ca. 120–160 meq O_2_/kg oil). It should be noted that both the GS used and the inlet/outlet drying temperatures were similar to the present work.

In addition to the PV, alpha-, beta-, delta- and gamma-tocopherols were quantified in the microcapsules during storage (see Figure S1 in the Supplementary Material). A significant decrease (*p* < 0.05) was observed in the content of alpha-, delta- and gamma-tocopherols for the microcapsules when compared to the fresh oil. This indicates the formation of radicals during emulsification and drying processes and that tocopherols were consumed as a consequence of their radical scavenging activity. It is worth mentioning that the high initial content of tocopherols in the fresh fish oil is a consequence of the addition of these compounds to the oil by the producer. The tocopherol content of the microencapsulates slightly changed over the storage time, regardless of the encapsulating agent used and the storage temperature. The latter leads us to conclude that the oxidative stability of the microcapsules during storage was not influenced by their tocopherol content but was mainly determined by the protection provided by the encapsulating agents, as well as the stabilization provided with the WPH. 

##### Secondary Volatile Oxidation Products (SVOP)

**Figure 3:**
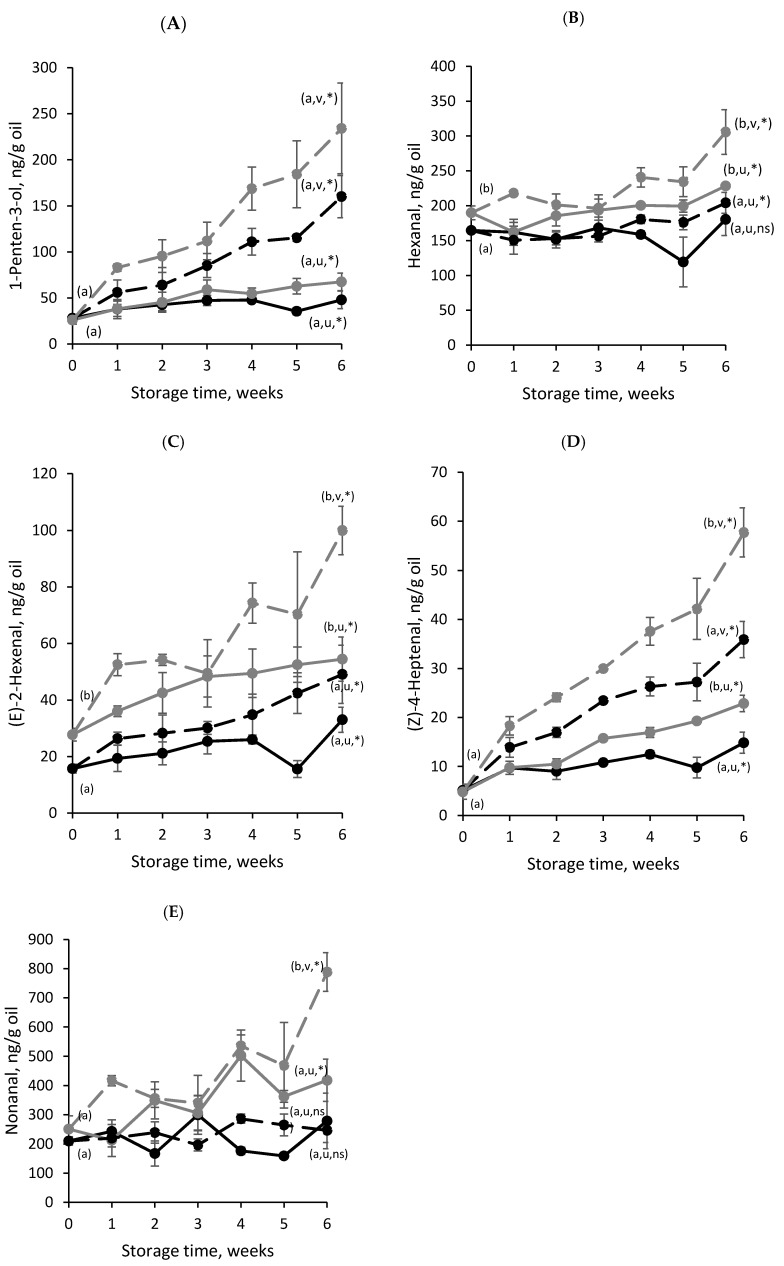
Secondary volatile oxidation products (1-penten-3-ol (**A**), hexanal (**B**), (**E**)-2-hexenal (**C**), (Z)-4-heptenal (**D**) and nonanal (**E**)) of spray-dried microcapsules loaded with fish oil during storage at: 4 °C (solid line, —•—) and 25 °C (broken line, - -•- -) encapsulated with glucose syrup (black) and maltodextrin (grey). Means within the same sampling point followed by a letter, a-b, indicates statistical differences (*p* ≤ 0.05) between encapsulating agents for the same storage temperature. Means within the same sampling point followed by a letter, u-v, indicates statistical differences (*p* ≤ 0.05) between storage temperatures for the same encapsulating agent. Means within the same sample followed by an asterisk (*) indicates statistical differences (*p* ≤ 0.05) between week 0 and week 6. Means within the same sample followed by “ns” indicates no statistical differences (*p* > 0.05) between week 0 and week 6.

Figure 3 shows the content of the SVOP (1-penten-3-ol, hexanal, (E)-2-hexenal, (Z)-4-heptenal and nonanal) in the microcapsules during storage. Despite the fact that hexanal and nonanal are compounds mainly derived from the oxidation of omega-6 and omega-9 fatty acids, these compounds have also been identified in oxidized fish oil. The rest of the identified SVOP (1-penten-3-ol, (E)-2-hexenal and (Z)-4-heptenal) are typical secondary oxidation products derived from omega-3 PUFAs, and all are related to undesirable odors and flavors [38]. For instance, the odor of 1-penten-3-ol, hexanal and (E)-2-hexenal have been described as earthy and grassy, while (Z)-4-heptenal and nonanal have been described as creamy and tallowy, respectively [39,40]. The odor threshold values for these compounds in oxidized oils range from 0.04 to 3.0 ppm [38].

**Figure 4:**
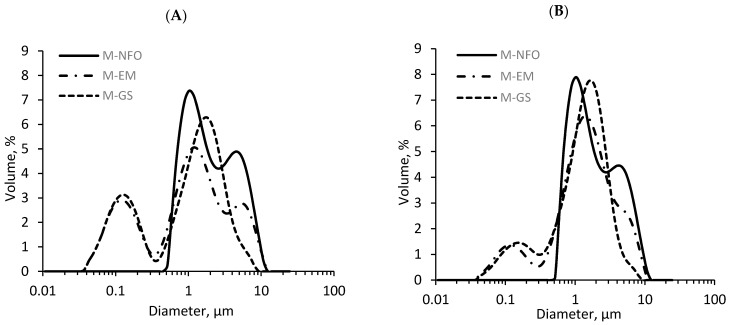
Droplet size distribution of low-fat mayonnaise enriched with: (i) neat fish oil (M-NFO), (ii) emulsified fish oil (M-EM) and (iii) microencapsulated fish oil (M-GS) at (**A**) day 0 and (**B**) day 28.

As occurred with the PV, the higher storage temperature favored lipid oxidation for both types of microencapsulates, since the highest SVOP content was found in the samples stored at 25 °C compared to those stored at 4 °C. However, different trends in the SVOP curves can be observed during the storage time (Figure 3). In the case of 1-penten-3-ol (Figure 3A) and (*Z*)-4-heptenal (Figure 3D), the influence of the storage temperature predominated over the wall material regarding lipid oxidation, since the lower contents of these compounds were found in the encapsulates stored at 4 °C, regardless of the encapsulating agent. Nonetheless, it is noteworthy that, in both cases, the GS-based microcapsules were the less-oxidized after six weeks of storage (48.06 ± 9.57 ng of 1-penten-3-ol/g oil and 14.85 ± 2.14 ng of (*Z*)-4-heptenal/g oil). On the contrary, the trend of hexanal (Figure 3B), (*E*)-2-hexenal (Figure 3C) and nonanal (Figure 3E) curves are in line with the PV results (Section 3.2.3, with MD21-based microcapsules presenting higher contents of the these volatiles independently of the storage temperatures when compared to GS-based microcapsules. This indicates, for the latter SVOP, a clear influence of the wall material over the storage temperature on microencapsulated fish oil oxidative stability. 

Most of the studies published regarding the oxidative stability of microencapsulated fish oil by spray-drying have only analyzed the course of propanal during storage, since it is an important SVOP resulting from the oxidation of both EPA and DHA [8,35,37,41]. However, our results show that different trends can be observed when studying more than one SVOP. Likewise, García-Moreno et al. [19] studied the course of 1-penten-3-ol, (E)-2-pentenal, heptanal and nonanal during 21 days of storage at 20 °C for electrosprayed microcapsules loaded with fish oil (20 wt%) into a matrix of pullulan blended with dextran or GS. For 1-penten-3-ol, the authors reported values ranging from 500–2000 ng/g oil, while, for nonanal, the values ranged from 2000–5000 ng/g oil. In any case, the values reported by these authors were much higher than those of the current work, which are well below the compounds‘ odor threshold limits in oil, indicating that the microcapsules may be stable in regard to their organoleptic properties. 

Taken altogether, the PV and SVOP content results show that the most oxidatively stable microcapsules were those produced with GS as a wall material. This is directly related to the lower molecular weight of GS as a result of its higher DE when compared to MD21, which led to microencapsulates: (i) with higher EE and retention of the core material and (ii) less porous and, thus, less permeable to prooxidant species (e.g., oxygen). However, the higher oxidative stability of the GS-based microencapsulates reported in the current study cannot only be attributed to the protective effect of the wall material. Tamm et al. [16] produced microcapsules (ca. 31 wt% oil load) spray-drying emulsions stabilized with β-LG hydrolysates (2.2 wt%) and GS as wall material. The authors reported a higher oxidative stability (based on the PV) of the microencapsulates containing hydrolysates when compared to those containing unmodified protein, which clearly revealed the additional protective effect of using whey protein hydrolysates for microencapsulating fish oil. These results are in line with those of our study, where the content of both the hydroperoxides (PV) and SVOP of GS-based microcapsules containing WPH as a film-forming material remained low during six weeks of storage. The protective effect of the WPH used in this study is related to its high emulsifying and antioxidant activities (both radical scavenging, EC_50_ = 4.45 ± 0.00 mg/mL, and metal chelating, EC_50_ = 0.95 ± 0.01 mg/mL), as reported in our previous work (unpublished results). The antioxidant activity of the WPH is mainly attributed to its high content in Tyr, Met and, to a lesser extent, His. Tyr has been related to possess radical scavenging activity due to the capacity of the phenolic group to serve as the hydrogen donor [12,42], Met reduces lipid hydroperoxides to the non-reactive species by oxidation to Met sulfoxide [12,43] and His possesses both radical scavenging and chelating activity [44]. Moreover, the small peptides produced as a result of protein hydrolysis (ca. 50% of the peptides between 0.5–3 kDa, (unpublished results) could have also improved the encapsulating matrix protective effect by acting as copolymers or fillers of the wall, thus limiting prooxidant diffusivity to a higher extent. This fact combined with an already low permeable wall matrix (e.g., GS) and low storage temperatures resulted in long-term oxidatively stable microcapsules. 

Hence, GS-based microcapsules loaded with fish oil and containing WPH as a film-forming material were further evaluated as an omega-3 delivery system to produce fortified low-fat mayonnaise.

### 3.3. Physical and Oxidative Stabilities of Fortified Low-Fat Mayonnaise 

The feasibility of using fish oil-loaded microcapsules produced by spray-drying (using GS as the encapsulating agent and WPH as the film-protein material) to enrich low-fat mayonnaise was evaluated. For this purpose, the physical and oxidative stabilities of low-fat mayonnaise samples fortified with: (i) neat fish oil (M-NFO), (ii) emulsified fish oil (M-EM) and (iii) microencapsulated fish oil (M-GS) were investigated.

#### 3.3.1. Physical Stability: Droplet Size Distribution and Viscosity 

Droplet size distributions of the mayonnaise samples indicate their physical stability but also the specific surface area, which influences lipid oxidation in the system. After production (day 0), the mayonnaise enriched with neat fish oil (M-NFO) showed a bimodal droplet size distribution with a main peak centered in ca. 1 µm and a second one, less representative, centered in ca. 4.7 µm (Figure 4A). Likewise, the mayonnaise sample containing microencapsulated fish oil (M-GS) showed a bimodal droplet size distribution with the peaks centered in 0.13 and 1.8 µm, respectively (Figure 4A). When comparing this curve to that of the emulsion used for the production of the microencapsulates, the droplet size of the first peak of the mayonnaise is in agreement with the peak of the monomodal curve of the emulsion fed to the spray-drier (ca. 0.13 µm, data not shown). Therefore, the first peak of the M-GS sample represents the droplet size of the fish oil, while the second peak is a consequence of the sunflower oil droplets dispersed during the mayonnaise production. The mayonnaise fortified with emulsified fish oil (M-EM) showed a trimodal droplet size distribution with the first peak overlapping with the M-GS sample (ca. 0.13 µm) and the other two peaks centered as those of the M-NFO sample (ca. 1 and 4.7 µm, respectively) (Figure 4A). The latter suggests that the fish oil emulsion maintained its physical integrity during the mayonnaise production process, despite the shear forces produced by the blender, and did not modify the sunflower oil droplet size distribution.

Table 2 shows that the mayonnaise sample containing neat fish oil (M-NFO) presented D[4,3] and D[3,2] values significantly higher (*p* < 0.05) than the rest of the samples at day 0. This is because, for the M-EM and M-GS samples, the fish oil was emulsified using a high-pressure homogenizer prior to the addition to the mayonnaise, which resulted in a lower size of the fish oil droplets when compared to the complete homogenization of the sunflower and fish oils while using the kitchen blender. It should be noted that, for the M-GS sample, the microcapsules were dispersed for 45 s into an already produced mayonnaise, which could have led to a further decrease of the size of the bulk droplets (sunflower oil droplets), explaining the lower D[4,3] values observed for this sample. Contrary to other studies, which found that the fortified mayonnaise with microencapsulated fish oil was the one with a higher D[4,3] value [24,45], our results may indicate that the capsules were intact and well-dispersed in the mayonnaise matrix at day 0. It should be noted that the differences in the D[4,3] values between our results and those reported in the previous studies mentioned [24,45] are mainly attributed to the different equipment used for mayonnaise production.

After 28 days of storage, fortified mayonnaise samples retained their physical integrity, since no phase separation or creaming were observed. However, changes in the droplet size distribution occurred (Figure 4B and Table 2). Contrary to the M-EM and M-GS samples, the D[4,3] and D[3,2] values of M-NFO decreased after 28 days of storage, suggesting that right after mayonnaise production, oil floccules were present, which may have disintegrated during storage [24]. For the M-EM and M-GS samples, a significant increase in the D[3,2] value suggested the coalescence or flocculation of oil droplets during storage caused by: (i) partial physical destabilization of the fish oil-in-water emulsion (in the case of the M-EM sample) or (ii) partial disintegration of the encapsulating wall, resulting in oil release (in the case of the M-GS sample) when these delivery systems were added to the food matrix.

All fortified low-fat mayonnaise samples presented pseudoplastic behaviors (see Figure S2 in the Supplementary Material). Table 2 shows the apparent viscosity values at a shear rate of 10 s^−1^ for the mayonnaise samples. It was observed that the M-GS presented a significantly higher apparent viscosity, followed by the M-EM and M-NFO mayonnaises. These results are in line with those previously reported by Miguel et al. [24], who attributed the higher viscosity of the mayonnaise sample enriched with zein-based fish oil microencapsulates to the intact microcapsules’ thickening effect in the aqueous phase. Moreover, it has been stated that the larger the oil droplet size, the lower the apparent viscosity, since a reduced surface-to-volume ratio of the disperse phase leads to less friction between the droplets [46]. The latter correlates well with these results, since as the droplet size of the fortified mayonnaise samples increased (e.g., higher for the M-NFO), their apparent viscosity decreased (e.g., lower for the M-NFO) (Table 2). After 28 days of storage, the apparent viscosity of the M-EM and M-GS samples did not change significantly (*p* > 0.05), contrary to the M-NFO sample. This may be explained by the presence of WPH in the M-EM and M-GS mayonnaises, the latter also containing GS. This fact resulted in an improved physical stability of the M-EM and M-GS samples, which was attributed to an increased viscosity [47].

Overall, the GS-based enrichment system led to a fortified mayonnaise with a high physical stability caused by an increased viscosity.

#### 3.3.2. Oxidative Stability of the Fortified Mayonnaise

##### Peroxide Value (PV)

**Figure 5:**
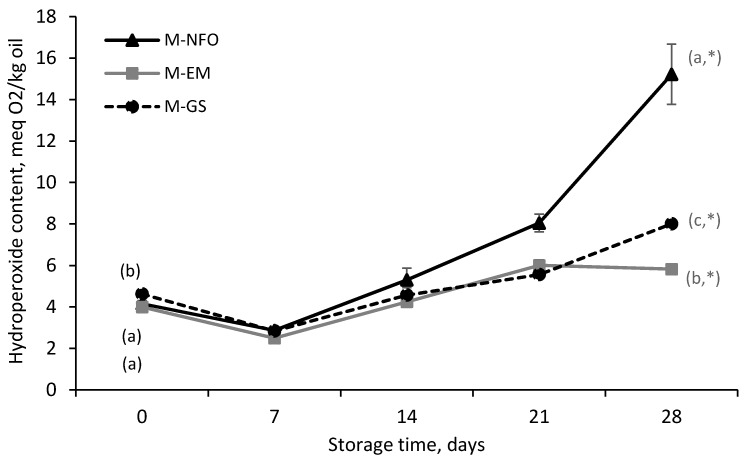
Peroxide value (PV) of low-fat fortified mayonnaise enriched with neat fish oil (M-NFO), emulsified fish oil (M-EM) or microencapsulated fish oil (M-GS) over storage time at 25 °C. Means within the same sampling point followed by a letter, a-c, indicates statistical differences (*p* ≤ 0.05). Means within the same sample followed by an asterisk (*) indicates statistical differences (*p* ≤ 0.05) between day 0 and day 28.

Figure 5 shows the evolution of the PV for the enriched low-fat mayonnaise samples during storage. It was observed that the PV at day 0 was similar among the three mayonnaise samples (4.0 ± 0.2 to 4.6 ± 0.1 meq O_2_/kg oil), despite the different degrees of initial oxidation of the enrichment systems: (i) NFO (PV = 1.3 ± 0.4 meq O_2_/kg oil; AV = 6.6 ± 0.7), (ii) fish oil-in-water emulsion stabilized with WPH (PV = 4.8 ± 1.2 meq O_2_/kg oil; AV = 6.2 ± 1.1) and (iii) fish oil-loaded glucose syrup microcapsules containing WPH as a film-forming material (PV = 7.0 ± 1.0 meq O_2_/kg oil; AV = 11.9 ± 1.5). This can be explained, because the majority of the oil present in the mayonnaise was SFO (PV = 3.5 ± 0.2 meq O_2_/kg oil; AV = 8.1 ± 1.1), which is more oxidatively stable than fish oil and may have masked the contribution of fish oil oxidation in the mayonnaise samples after production.

Over storage time, the PV course showed a lag phase of 1 week for the three enriched mayonnaise samples (Figure 5). However, from week 2 onwards, the PV of the three samples assayed increased progressively to final values significantly different (*p* < 0.05) from those of the beginning of the storage time. Interestingly, at day 28, the mayonnaise fortified with NFO presented a significantly higher PV (15.22 ± 1.45 meq O_2_/kg oil) compared to M-GS and M-EM. It should be also noted that, although the final PV of M-GS was significantly higher than the PV of M-EM, a slight decrease in the PV of M-EM was observed after day 21. This indicates that the rate of hydroperoxide decomposition was higher compared to the rate of hydroperoxide formation after day 21 for M-EM.

It is also noteworthy that our results are not aligned with those reported by Hermund et al. [45]. These authors found a lower oxidative stability for low-fat mayonnaise enriched with GS-based electrosprayed microcapsules (PV = 8–12 meq O_2_/kg oil) when compared to low-fat mayonnaise enriched with NFO (PV = 2 meq O_2_/kg oil) after 28 days storage at 25 °C. This finding was attributed to the partial disintegration of the hydrophilic biopolymer-based wall matrix of the electrosprayed microcapsules during mayonnaise production, which led to the release of already oxidized fish oil. On the other hand, Miguel et al. [24] produced fortified mayonnaise enriched with zein-based electrosprayed microcapsules with a high oxidative stability during storage (PV = 2 meq O_2_/kg oil after 21 days of storage at 25 °C). In the latter work, the authors confirmed that the microencapsulates remained intact after mayonnaise production, which explained the enhanced oxidative stability of the fortified product. Taking both studies’ results into account leads us to speculate that, in the current study, the physical integrity of the encapsulating wall was not severely affected either by the fortified mayonnaise production process (high-speed blending to disperse the capsules) nor the food matrix (water-based emulsion). However, the latter requires further investigation.

##### P-Anisidine Value (AV)

**Figure 6:**
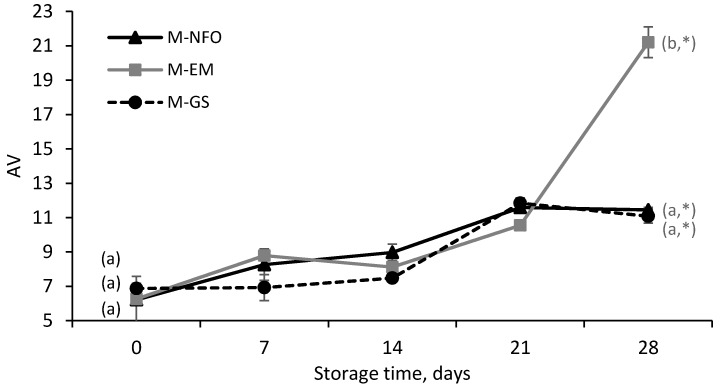
P-anisidine value (AV) of low-fat fortified mayonnaise enriched with neat fish oil (M-NFO), emulsified fish oil (M-EM) or microencapsulated fish oil (M-GS) over storage time at 25 °C. Means within the same sampling point followed by a letter, a-c, indicates statistical differences (*p* ≤ 0.05). Means within the same sample followed by an asterisk, *, indicates statistical differences (*p* ≤ 0.05) between day 0 and day 28.

The p-anisidine value (AV) indicates the formation of secondary oxidation products, principally 2-alkenals and 2,4-alkadienals, which arise as a consequence of decomposition of the primary oxidation products (hydroperoxides) [48]. The initial AV of the fortified mayonnaises ranged from 6.2 ± 1.4 to 6.9 ± 0.3, with no significant differences among the samples (*p* > 0.05) (Figure 6).

During storage, the AV increased progressively for all the samples until day 21, from which a sharp increase in only the M-EM sample occurred. These results are in line with those previously reported for PV since, after day 21, the hydroperoxide content of the M-EM sample decreased (Figure 5), suggesting the appearance of secondary oxidation products. The poorer oxidative stability of the M-EM sample could be result of the emulsification process itself (production of a coarse emulsion and subsequent homogenization). The first step involves intense mechanical stress, which favors air inclusion, while the latter disrupts/rearranges the oil droplets, favoring a better distribution of the prooxidant species within the matrix (e.g., oxygen or metal ions) [8]. Moreover, high-pressure homogenization causes a significant increase in a specific surface area, as denoted by the lower D[4,3] values of M-EM when compared to M-NFO (Table 2), which results in a larger surface of contact between the oil and prooxidants. Likewise, Let at al. [49] reported a lower oxidative stability (based on the content of 2-hexenal, 4-heptenal and 2,4-heptadienal during storage) for a salad dressing enriched with emulsified fish oil when compared to the addition of neat fish oil. The authors also reported that the oxidative stability of neat fish oil-fortified yogurt was higher than that of the yogurt fortified with emulsified fish oil.

After 28 days of storage, no significant differences were observed in the AV when comparing the M-NFO and M-GS samples (*p* > 0.05). However, M-GS showed a lag phase of one week, contrary to M-NFO (Figure 6), which could suggest a protective effect of the microencapsulates against fish oil oxidation during the early stages of storage. In this line, Nielsen and Jacobsen [50] investigated the fish oil-enrichment approach for a fish oil-fortified cod pâté and reported that the addition of microencapsulated fish oil resulted in a better oxidative stability after storage (based on 1-penten-3-oil content) than the samples fortified either with emulsified fish oil or neat fish oil.

Taken altogether, the PV and AV results indicate that the most oxidatively stable mayonnaise sample after one month of storage was M-GS, closely followed by M-NFO, while M-EM showed the lowest oxidative stability. Hence, the enhanced protection against lipid oxidation could be the result of the maintenance of the physical integrity of the encapsulating wall during mayonnaise production, which provides a physical barrier limiting the contact between fish oil droplets and prooxidants. Moreover, the higher apparent viscosity of M-GS as a result of the thickening effect of the microencapsulates may have also limited prooxidant species diffusivity, reducing lipid oxidation [51].

Our findings revealed that the use of WPH as a film-forming material, besides its metal chelating activity, which is of special interest to reduce lipid oxidation in egg yolk-based products [45], was only beneficial for the production of microcapsules but not emulsions to be used as omega-3 delivery systems. Hence, the processing stages required to obtain the fish oil-loaded microcapsules containing WPH as a film-forming material (e.g., homogenization and spray-drying) are justified, since they result in an omega-3 delivery system that better protects the fish oil when it is incorporated into low-fat mayonnaise.

## 4. Conclusions

Oxidatively stable microcapsules loaded with fish oil (ca. 13 wt%) were produced via spray-drying using either GS or MD21 as wall materials and WPH as a film-forming material. GS-based microcapsules showed a higher oxidative stability when compared to MD21-based microcapsules at the two storage temperatures assayed (4 and 25 °C), which was mainly attributed to the lower oxygen diffusivity in GS-microcapsules. However, the higher oxidative stability for the GS-based microcapsules obtained, when compared to previous studies, also suggested a positive influence of the use of WPH as a film-forming material exhibiting antioxidant activity. Moreover, our results showed that the delivery system used to enrich low-fat mayonnaise played a major role on the oxidative stability of the fortified product. Adding the fish oil in the form of a fish oil-in-water emulsion stabilized with WPH-favored lipid oxidation in the enriched product when compared to the addition of either neat or microencapsulated fish oil. This may be explained by successive emulsification processes that contributed to dispersing oxygen and prooxidant agents within the matrix, as well as to increasing the interfacial area. Fortified mayonnaise with GS-based microcapsules loaded with fish oil and containing WPH as a film-forming material showed the higher oxidative stability after storage. This was mainly explained because the physical integrity of the microencapsulates may have remained intact after mayonnaise production. Thus, our results show the potential of using WPH as a film-forming material for the production of fish oil-loaded microcapsules to be used as omega-3 delivery systems.

## Figures and Tables

**Table 1:** Oil droplet size distribution (ODSD) of fresh and reconstituted emulsions during storage time at 4 and 25 °C.

d_90_, µm			
-		GS	MD21
**Parent emulsions**		0.587 ± 0.001 ^+,*^	0.555 ± 0.001 ^+,*^
**Reconstituted emulsion after spray-drying**			
Week 0		0.613 ± 0.006 ^+,a,u,*^	0.663 ± 0.001 ^+,a,u,*^
4 °C	Week 2	0.624 ± 0.006 ^a,j,*^	0.699 ± 0.005 ^b,j,*^
	Week 4	0.653 ± 0.002 ^b,j,*^	0.730 ± 0.001 ^c,j,*^
	Week 6	0.654 ± 0.004 ^b,j,*^	0.825 ± 0.001 ^d,j,*^
25 °C	Week 2	0.599 ± 0.001 ^u,k,*^	0.703 ± 0.011 ^v,j,*^
	Week 4	0.669 ± 0.005 ^v,j,*^	0.717 ± 0.003 ^v,k,*^
	Week 6	0.659 ± 0.010 ^v,j,*^	0.882 ± 0.002 ^w,k,*^

GS: glucose syrup and MD21: maltodextrin with DE 21. Means within the same column followed by a plus sign, ^+^, indicates statistical differences (*p* ≤ 0.05) between the parent and reconstituted (week 0) emulsions. Means within the same column followed by different letters, ^a–c^, indicate statistical differences (*p* ≤ 0.05) between sampling points for the same encapsulating agent at 4 °C. Means within the same column followed by different letters, ^u–w^, indicate statistical differences (*p* ≤ 0.05) between sampling points for the same encapsulating agent at 25 °C. Means within the same column followed by different letters, ^j,k^, indicate statistical differences (*p* ≤ 0.05) between samples stored at different temperatures for the same encapsulating agent at the same sampling point. Means within the same row followed by an asterisk, *, indicates statistical differences (*p* ≤ 0.05) between encapsulating agents.

**Table 2:** Droplet size and apparent viscosity (γ = 10 s^−1^) of fortified low-fat mayonnaise enriched with neat fish oil (M-NFO), emulsified fish oil (M-EM) or microencapsulated fish oil (M-GS).

	Droplet Size				Apparent Viscosity	
	Day 0		Day 28		(γ = 10 s^−1^), Pa·s	
Sample	D[3,2], µm	D[4,3], µm	D[3,2], µm	D[4,3], µm	Day 0	Day 28
M-NFO	1.555 ± 0.020 ^a^	2.724 ± 0.035 ^a^	1.487 ± 0.006 ^a,*^	2.553 ± 0.011 ^a,*^	2.6 ± 0.3^a^	2.0 ± 0.1 ^a,*^
M-EM	0.310 ± 0.008 ^b^	1.865 ± 0.052 ^b^	0.516 ± 0.038 ^b,*^	1.985 ± 0.045 ^b,ns^	4.8 ± 0.1^b^	5.1 ± 0.5 ^b,ns^
M-GS	0.302 ± 0.001 ^b^	1.436 ± 0.039 ^c^	0.519 ± 0.027 ^b,*^	1.538 ± 0.068 ^c,ns^	9.7 ± 0.3^c^	9.4 ± 0.2 ^c,ns^

Means within the same column followed by a letter, ^a–c^, indicates statistical differences (*p* ≤ 0.05) between samples. Means within the same sample followed by an asterisk, *, indicates statistical differences (*p* ≤ 0.05) between day 0 and day 28. Means within the same sample followed by “^ns^” indicates no statistical differences (*p* > 0.05) between day 0 and day 28.

## Supplementary Materials

The following are available online at https://www.mdpi.com/2304-8158/9/5/545/s1: Figure S1: Tocopherol content of spray-dried capsules loaded with fish oil during storage at: 4 °C (solid line, —•—) and 25 °C (broken line, - -•- -) microencapsulated with glucose syrup (black) or maltodextrin (grey); Figure S2: Viscosity of mayonnaise enriched with: (i) neat fish oil (M-NFO), (ii) emulsified fish oil (M-EM) and (iii) microencapsulated fish oil (M-GS) at (A) day 0 and (B) day 28.
