# Supplementary material for: Development of Fish Oil-Loaded Microcapsules Containing Whey Protein Hydrolysate as Film-Forming Material for Fortification of Low-Fat Mayonnaise
Source: Foods. 2020 Apr 30;9(5):545. doi: 10.3390/foods9050545 (PMC7278592; doi:10.3390/foods9050545)
Supplement: Supplementary file 1 [file foods-09-00545-s001.pdf]

Supplementary material

(A)

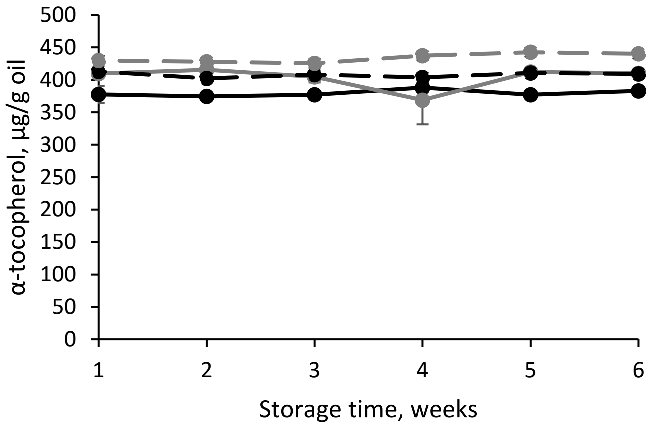

(B)

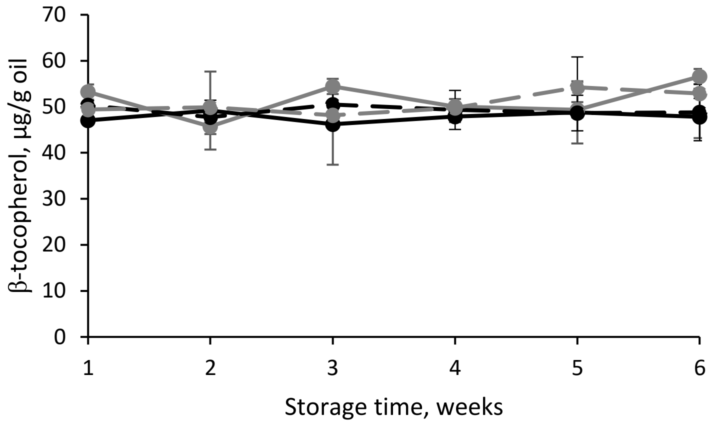

(C)

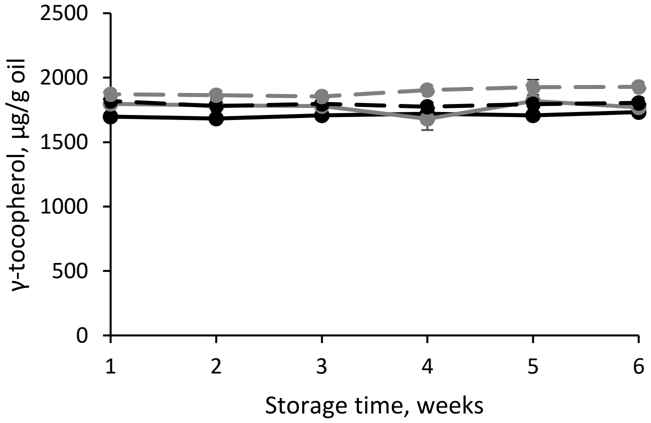

(D)

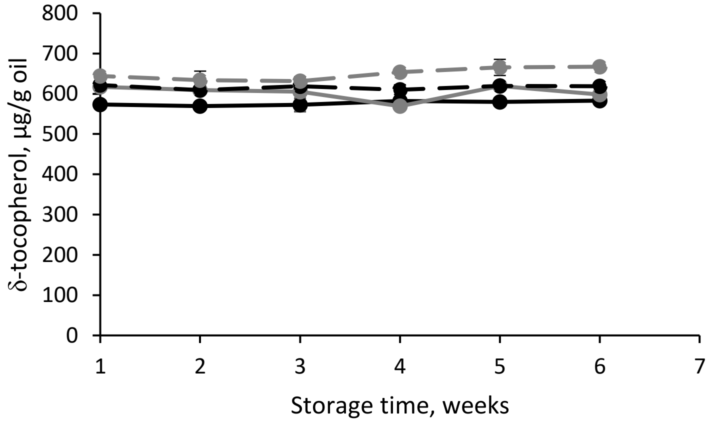

Figure 7. Tocopherol content of spray-dried capsules loaded with fish oil during storage at: 4 °C (solid line, —•—) and 25 °C (broken line, - -•- -) encapsulated with glucose syrup (black) or maltodextrin (grey).

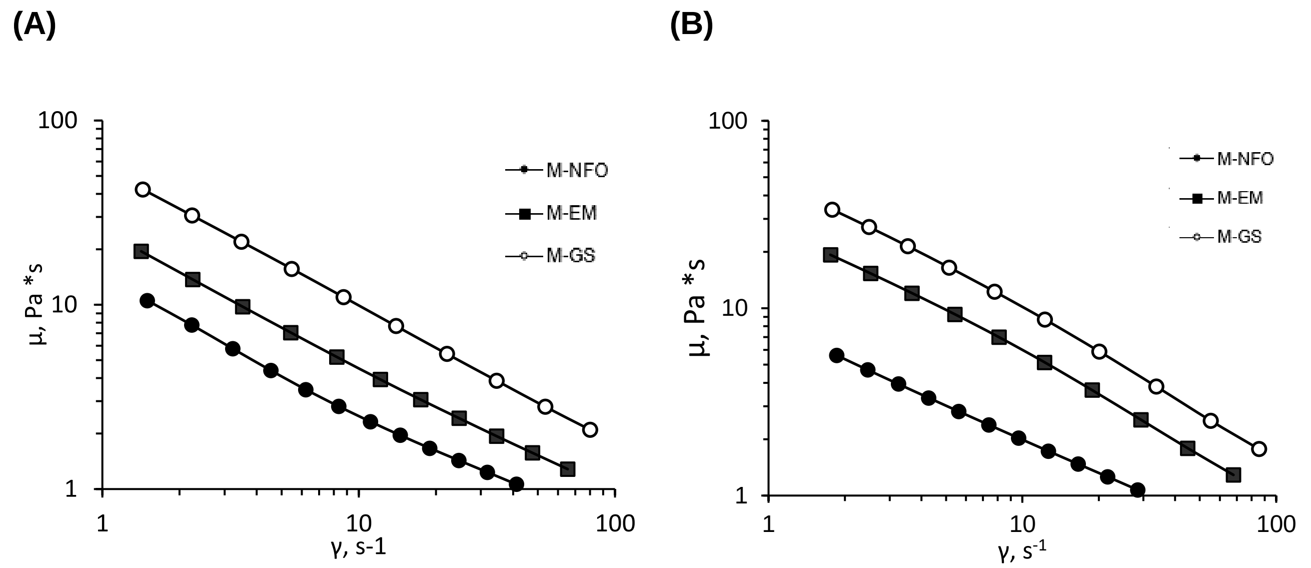

Figure 1. Viscosity of mayonnaise enriched with: i) neat fish oil (M-NFO), ii) emulsified fish oil (M-EM) and iii) encapsulated fish oil (M-GS) at (A) day 0 and (B) day 28.
